# Epidemiological significance of dengue virus genetic variation in mosquito infection dynamics

**DOI:** 10.1371/journal.ppat.1007187

**Published:** 2018-07-13

**Authors:** Albin Fontaine, Sebastian Lequime, Isabelle Moltini-Conclois, Davy Jiolle, Isabelle Leparc-Goffart, Robert Charles Reiner, Louis Lambrechts

**Affiliations:** 1 Insect-Virus Interactions Group, Department of Genomes and Genetics, Institut Pasteur, Paris, France; 2 Unité de Parasitologie et Entomologie, Département des Maladies Infectieuses, Institut de Recherche Biomédicale des Armées, Marseille, France; 3 Génomique Evolutive, Modélisation et Santé, Unité Mixte de Recherche 2000, Centre National de la Recherche Scientifique, Paris, France; 4 Aix Marseille Université, IRD, AP-HM, SSA, UMR Vecteurs–Infections Tropicales et Méditerranéennes (VITROME), IHU—Méditerranée Infection, Marseille, France; 5 KU Leuven Department of Microbiology and Immunology, Rega Institute, Laboratory of Clinical and Epidemiological Virology, Leuven, Belgium; 6 Centre National de Référence des Arbovirus, Institut de Recherche Biomédicale des Armées, Marseille, France; 7 Institute for Health Metrics and Evaluation, University of Washington, Seattle, Washington, United States of America; Imperial College London, UNITED KINGDOM

## Abstract

The kinetics of arthropod-borne virus (arbovirus) transmission by their vectors have long been recognized as a powerful determinant of arbovirus epidemiology. The time interval between virus acquisition and transmission by the vector, termed extrinsic incubation period (EIP), combines with vector mortality rate and vector competence to determine the proportion of infected vectors that eventually become infectious. However, the dynamic nature of this process, and the amount of natural variation in transmission kinetics among arbovirus strains, are poorly documented empirically and are rarely considered in epidemiological models. Here, we combine newly generated empirical measurements *in vivo* and outbreak simulations *in silico* to assess the epidemiological significance of genetic variation in dengue virus (DENV) transmission kinetics by *Aedes aegypti* mosquitoes. We found significant variation in the dynamics of systemic mosquito infection, a proxy for EIP, among eight field-derived DENV isolates representing the worldwide diversity of recently circulating type 1 strains. Using a stochastic agent-based model to compute time-dependent individual transmission probabilities, we predict that the observed variation in systemic mosquito infection kinetics may drive significant differences in the probability of dengue outbreak and the number of human infections. Our results demonstrate that infection dynamics in mosquitoes vary among wild-type DENV isolates and that this variation potentially affects the risk and magnitude of dengue outbreaks. Our quantitative assessment of DENV genetic variation in transmission kinetics contributes to improve our understanding of heterogeneities in arbovirus epidemiological dynamics.

## Author summary

Transmission of arboviruses by mosquitoes is a time-dependent process because dissemination of the virus from the insect’s digestive tract, where the infection is initially established, to the salivary glands, where it can be transmitted to the next host, typically takes several days. The duration of this time interval is an important determinant of the proportion of infected mosquitoes that live long enough to become infectious. Due to the lack of available data, epidemiological models rarely account for the dynamic nature of this process and its natural variation among arbovirus strains. In this study, we experimentally monitored the dynamics of mosquito infection by eight strains of dengue virus and found significant differences between them. In addition, we used the empirical data obtained in a simulation model to show that the differences observed among the strains are expected to result in human dengue outbreaks of different probability and size. Together, our results indicate that natural variation in mosquito infection dynamics between arbovirus strains may contribute to the unexplained heterogeneity of dengue virus transmission patterns.

## Introduction

In the last few decades, arthropod-borne viruses (arboviruses) have become major contributors of global mortality and disability, calling for an improved understanding of their transmission dynamics [1]. Transmission of arboviruses is governed by a complex set of biotic and abiotic factors [2], the majority of which relate to the vector biology [3]. Individual, temporal and spatial variability in these factors results in considerable heterogeneities in arbovirus transmission dynamics, but the relative contribution of each factor is still poorly understood [4–7].

In classic models of mosquito-borne pathogen transmission, one of the most influential parameters is the extrinsic incubation period (EIP), defined as the time interval between pathogen acquisition and pathogen transmission by the vector [8]. In these models, EIP interacts with the probability of mosquito survival as an exponential term, so that even a small deviation in EIP will substantially change the number of mosquitoes surviving long enough to become infectious. Despite the recognized importance of EIP as a powerful determinant of mosquito-borne pathogen transmission, variation in the duration of EIP has rarely been accounted for in models of mosquito-borne pathogen epidemiology [9]. Current approaches to assess the epidemiological significance of variation in arbovirus transmission kinetics rely on temperature-dependent models developed from limited empirical data [10–12]. These models generally assume that the only factor affecting transmission kinetics is ambient temperature.

Here, we investigated whether arbovirus genetic variation may alter transmission kinetics in epidemiologically significant ways. Specifically, we experimentally measured variation in transmission kinetics among several wild-type isolates of dengue virus (DENV) type 1 representing the worldwide genetic diversity of recently circulating strains. DENV are positive-sense, single-stranded RNA viruses in the *Flavivirus* genus that cause more human infections than any other arbovirus [13]. They exist as four genetically divergent types that loosely cluster antigenically [14]. Each DENV type is divided into major phylogenetic clades, often termed genotypes, which in turn contain a wide diversity of strains [15]. All four serotypes of DENV are mainly transmitted by the mosquito *Aedes aegypti* worldwide [16].

A mosquito’s intrinsic ability to transmit a virus, referred to as vector competence, is a stepwise procedure that begins when the mosquito takes an infectious blood meal on a viremic host. Following initial infection and replication in the midgut, the virus disseminates systemically from the midgut to secondary tissues including salivary glands, where it can be released into saliva during a subsequent blood meal. The probability of detecting DENV in *Ae*. *aegypti* saliva is correlated with the amount of virus that has spread out of the midgut [17], which is often used as a proxy for transmission potential. Although it is generally measured by end-point laboratory assays, vector competence is a dynamic process that combines with vector survival and EIP to determine the proportion of infectious vectors over time [18]. The duration of DENV EIP is in the range of 8–12 days at a temperature of 25–28°C [12, 19, 20], which is close to adult mosquito life expectancy under field conditions.

The influence of EIP on DENV epidemiology was recently examined in the context of *Wolbachia*-mediated blocking of virus transmission [21, 22]. Earlier studies suggested that viral genetic variation in EIP could also influence DENV epidemiology in nature. For example, differences of EIP among DENV type 2 strains have been proposed as a mechanism of genotype replacement and dengue emergence [23]. By comparing three DENV type 2 strains of the American genotype with three DENV type 2 strain of the Southeast Asian genotype, Anderson and Rico-Hesse estimated a 2- to 65-fold increase in vectorial capacity for the invasive Southeast Asian genotype relative to the resident American genotype [23]. This increase resulted from a 7-day difference in EIP between genotypes (7 days for the Southeast Asian vs. 14 days for the American genotype) across a range of daily survival rates. However, EIP was estimated as a single ‘threshold’ value defined as the time at which 50% of salivary glands became infected, which likely misrepresents EIP for an entire population [24].

To more accurately assess the epidemiological significance of DENV genetic variation in transmission kinetics, we used two complementary approaches. First, we experimentally monitored the cumulative proportion of mosquitoes with a systemic infection over time for eight genetically diverse, low-passage DENV type 1 isolates. We then used these *in vivo* empirical data to derive a 3-parameter model of the kinetics of systemic infection, a proxy for transmission potential, and compare transmission dynamics among DENV isolates. Second, we implemented a stochastic agent-based model accounting for intra-mosquito and intra-human viral dynamics to compute time-dependent individual transmission probabilities and simulate arbovirus outbreaks *in silico*. Using this model, we showed that the empirically observed differences in DENV transmission kinetics have the potential to significantly influence the probability and size of human dengue outbreaks.

## Results

### Diverse field-derived DENV isolates display variation in systemic infection kinetics

**Fig 1:**
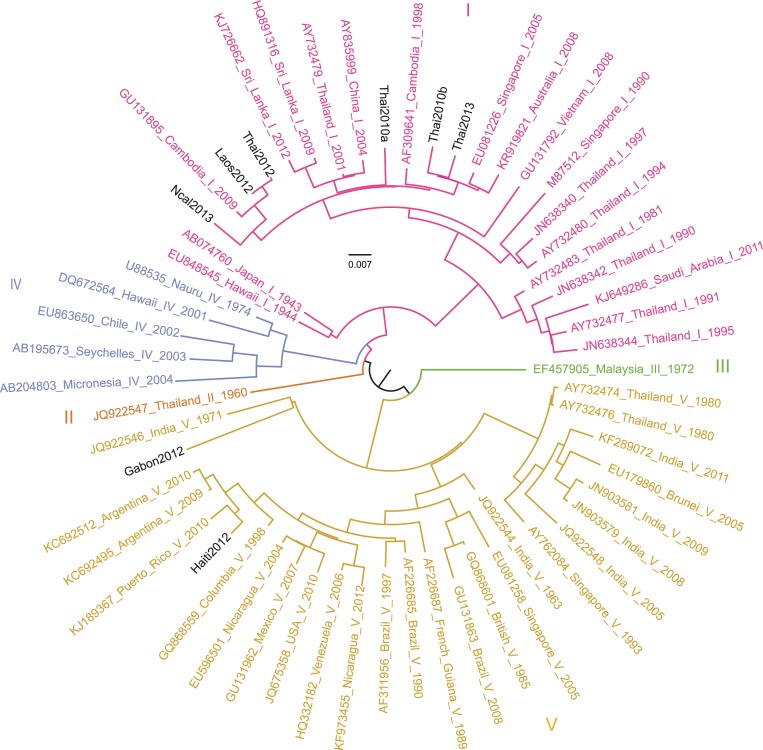
Phylogenetic relationships between DENV isolates of the study. Full-length genome sequences were used to infer the phylogenetic relationships among the isolates of the study in a background of representative sequences of DENV type 1. Genotypes are represented with distinct colors and the eight DENV isolates of the study are shown in black. Inferences were calculated with a maximum-likelihood method implemented in RAxML v.8.2.10[50]. The scale bar represents 0.007 substitutions per site.

We orally exposed wild-type *Ae*. *aegypti* mosquitoes to a standardized infectious dose of eight DENV type 1 isolates derived from human sera sampled in various endemic areas worldwide during 2010–2013 (Table 1). The eight DENV type 1 isolates were genetically diverse and included six isolates belonging to genotype I, and two isolates belonging to genotype V (Fig 1). Mosquitoes were collected 4, 6, 8, 12 and 18 days after virus oral exposure and assayed for DENV midgut and/or systemic infection. For each isolate, the mean number of mosquitoes assayed per time point was 25 and ranged from 10 to 32.

**Table 1:** Description of DENV type 1 isolates used in this study. The blood meal titer refers to the concentration of infectious viral particles (expressed in log_10_-transformed focus-forming units per mL) measured in the artificial blood meal offered to mosquitoes. The passage history refers to the number of prior amplifications in C6/36 cells.

DENV-1 isolate	Genotype	Geographical origin	Year of isolation	Blood meal titer (log_10_ FFU/mL)	Passage history	GenBank accession number
Thai2010a	I	Thailand	2010	5.74	5	HG316481
Thai2010b	I	Thailand	2010	5.70	6	HG316482
Thai2012	I	Thailand	2012	5.80	3	MG877554
Thai2013	I	Thailand	2013	5.80	2	MG877556
Laos2012	I	Laos	2012	5.85	3	MG877552
NCal2013	I	New Caledonia	2013	5.77	2	MG877555
Gabon2012	V	Gabon	2012	5.82	5	MG877557
Haiti2012	V	Haiti	2012	5.81	3	MG877553

We first tested infection status (S1 Fig) and subsequently focused on systemic infection dynamics of midgut-infected mosquitoes. Systemic DENV infection was determined at each time point based on virus presence in head tissues. Systemic infection prevalence among midgut-infected mosquitoes ranged from 4.5% to 100% across isolates and time points and was significantly influenced by the time point (*p* = 2.0×10^−16^) but not by uncontrolled variation in blood meal titers (*p* = 0.08) or the interaction between time point and blood meal titer (*p* = 0.61).

**Fig 2:**
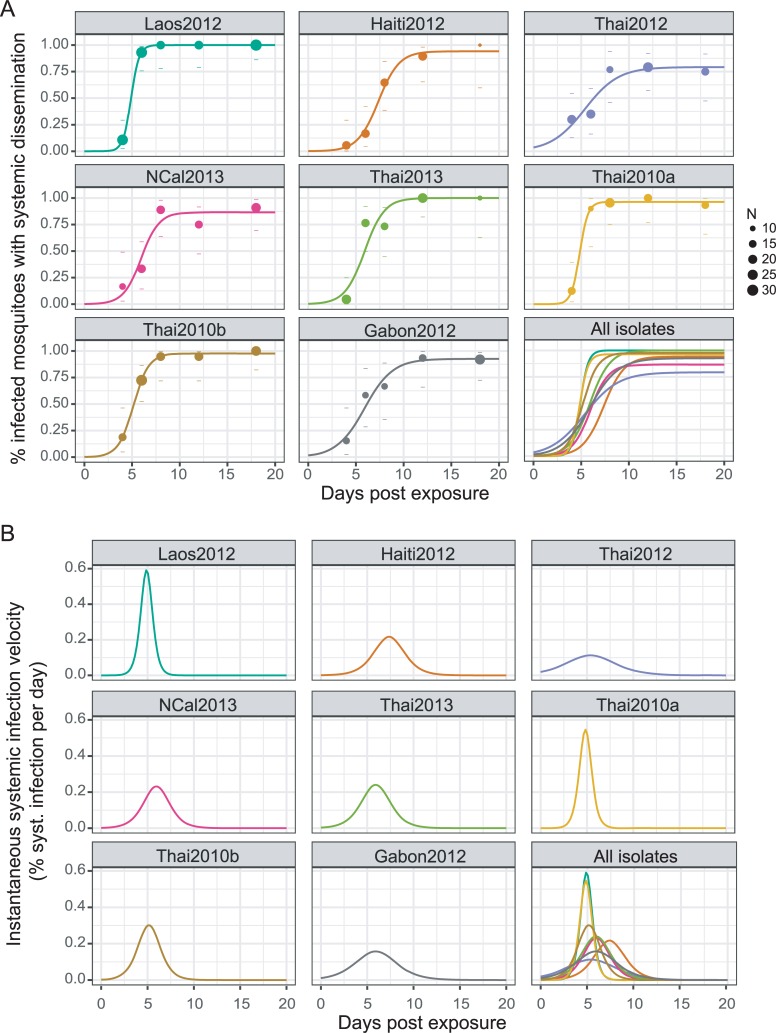
Variation in the kinetics of systemic mosquito infection between DENV isolates. (A) The cumulative prevalence of systemic infection over time post virus exposure is shown for each DENV isolate. Data points represent the observed prevalence at each time point with their size being proportional to the sample size (number of mosquitoes tested). Dashes represent the 95% confidence interval of the prevalence. Lines correspond to the fitted values obtained with a 3-parameter logistic model. (B) The daily rate of new systemic infections (instantaneous velocity) over time post virus exposure is shown for each DENV isolate. It was calculated as the first derivative of the cumulative systemic infection function and is equivalent to the frequency distribution of lag time values among individual mosquitoes. In both graphs, the lower right panel shows the merged fitted values for all isolates.

To quantify the kinetics of systemic DENV infection, we assumed that the cumulative change in the proportion of mosquitoes with a systemic DENV infection over time post exposure followed a sigmoid function. A logistic model (see Materials and Methods) was fitted to the empirical data to estimate three parameters (*K*, *B*, *M*) describing the kinetics of each isolate (Table 2; Fig 2A). *K* is the saturation level and represents the maximum proportion of infected mosquitoes with a systemic infection. *B* is the slope factor and represents the maximum slope of the cumulative function scaled by *K*. *M* is the lag time and represents the time at which the absolute increase in cumulative proportion of systemic infection is maximal, which is also when the systemic infection prevalence equals *K*/2.

**Table 2:** Parameter estimates of systemic infection kinetics for each DENV isolate. Parameter values for each isolate were inferred from the cumulative change in the proportion of mosquitoes with a systemic DENV infection over time post exposure using model optimization. *K* is the saturation level and represents the maximum proportion of mosquitoes with a systemic infection. *B* is the slope factor and represents the maximum slope of the cumulative function scaled by *K*. Δt is derived from *B* and represents the time required to rise from 10% to 90% of the saturation level. *M* is the lag time and represents the time at which the absolute increase in cumulative proportion is maximal, which is also when the systemic infection prevalence equals *K*/2. Background shading (grey scale) indicates the best isolate groupings as determined by the AIC method.

Isolate	*K* (%)	*B* (% day^-1^)	Δt (days)	*M* (days)
Gabon2012	93	68	6.45	5.92
NCal2013	87	107	4.11	5.96
Thai2012	79	57	7.73	5.37
Haiti2012	94	92	4.78	7.36
Thai2010b	98	124	3.55	5.15
Thai2013	100	96	4.57	5.92
Thai2010a	96	227	1.94	4.84
Laos2012	100	237	1.85	4.90

Among isolates, the saturation level *K* ranged from 79% to 100% (mean ± SD: 93 ± 7%). The slope factor *B* was transformed into Δt, the time required to rise from 10% to 90% of the saturation level, which ranged from 1.85 to 7.73 days (mean ± SD: 4.37 ± 2.03 days). The lag time *M* ranged from 4.84 to 7.36 days (mean ± SD: 5.68 ± 0.82 days). There was no statistically significant correlation between the three parameters (S2 Fig). However, isolates with a high saturation level tended to display a large slope factor and a short lag time. To graphically represent EIP variation among DENV isolates, we used the distribution of lag time (*M*) values as a proxy. We obtained the daily rate of new systemic infections by calculating the first derivative of the cumulative systemic infection function (Fig 2B). The curve of this rate over time is equivalent to the distribution of lag time values among individual mosquitoes.

**Fig 3:**
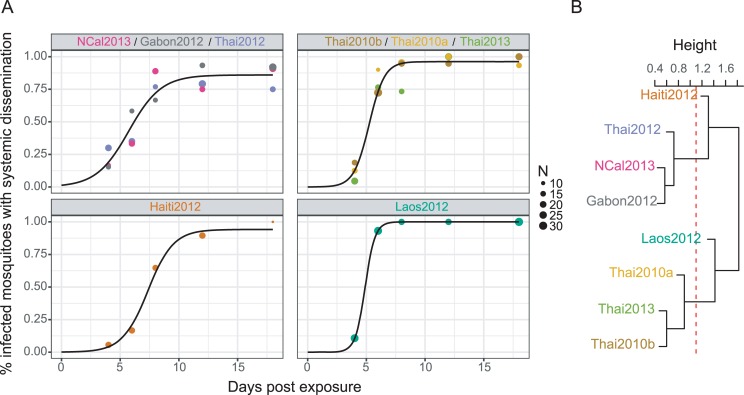
Comparison of systemic mosquito infection kinetics among DENV isolates. A logistic 3-parameter model was fitted to all permutations of isolates, with isolates from the same group being forced to share the same parameters. Panel (A) represents the isolate grouping that maximizes the probability function for systemic infection dynamics. This isolate grouping had the lowest AIC value among all tested permutations (S5 Fig). For each isolate group, data points represent the empirical measurements color-coded by isolate and the black line is the logistic fit of the group. An affinity matrix among isolates was derived from AIC values obtained from all model comparisons (S5 Fig). Panel (B) shows the dendrogram obtained from a hierarchical clustering analysis performed on the affinity scores. The distance between isolates corresponds to the number of times two isolates shared the same groupings with respect to their AIC. The red dotted line shows the cut-off value leading to the best AIC-based isolate grouping.

To quantitatively compare the kinetics of systemic infection among DENV isolates, we fitted a global likelihood model based on the 3-parameter logistic function to the empirical data for all combinations of permutations of isolates (see Materials and Methods). Based on Akaike Information Criterion (AIC) values, we identified four groups of isolates (Fig 3B and 3C). The first group had slow systemic infection dynamics and included three DENV isolates (Gabon2012, NCal2013, Thai2012) with a relatively low saturation level, small slope factor and long lag time. The second group had fast systemic infection dynamics and included three DENV isolates (Thai2010b, Thai2013, Thai2010a) with a higher saturation level, larger slope factor and shorter lag time. The last two groups consisted of one DENV isolate each (Laos2012 and Haiti2012) that had unique combinations of parameters (Table 2). A hierarchical clustering analysis performed on the affinity matrix derived from AIC scores of all combinations of isolates revealed that isolate Laos2012 branched with the fast-disseminating infection group whereas isolate Haiti2012 branched with the slow-disseminating infection group (Fig 3C). The groups did not relate to DENV type 1 genotypes, as for example Haiti2012 and Gabon2012 (both from genotype V) did not group together.

The hierarchical clustering results for midgut infection prevalence did not match the results obtained for systemic infection prevalence, indicating that midgut infection patterns are poor predictors of differences in the kinetics of systemic infection. Likewise, there was no association between the viral phylogeny and the kinetics of systemic infection (S3 Fig). As expected with such a small number of isolates, there was no statistically significant association between individual DNA polymorphisms or amino-acid differences with any of the three parameters describing the kinetics of systemic infection.

### Empirically observed variation in DENV transmission kinetics has the potential to dramatically impact the risk and magnitude of dengue outbreaks

To assess the epidemiological significance of the differences observed in the kinetics of DENV systemic infection in mosquitoes, we implemented a stochastic agent-based model that accounted for variation in the three parameters (*K*, *B*, *M*) of the logistic model described above, and their effect on the probability and size of dengue outbreaks. This method allows individual humans and mosquitoes, each one being characterized by a unique set of infection parameters, to interact within a simulated environment, and was designed to model the unfolding of real-world arboviral epidemics (see Materials and Methods). The 3-parameter function that was used to model the kinetics of systemic mosquito infection was incorporated as a time-dependent probability function to determine transmission status when a contact occurred between a human and a mosquito. Starting with one infected human in a population of 10,000 individuals, the model was run 100 independent times over 400 days using different combinations of the three parameters in the same range as the empirical values. The total number of infected humans was recorded for each combination of realistic parameter values. All combinations of parameter values resulted in a certain proportion of outbreaks without any secondary human infection (S4 File). Outbreaks with ≥1 secondary human infections were mainly partitioned into small-scale outbreaks (<100 secondary infections) and large-scale outbreaks (≥100 secondary infections) leading to a strongly bimodal distribution of outbreak size (S4A Fig). Lag time (*M*) had the most significant effect on the initiation of outbreaks (S4B and S4C Fig).

**Fig 4:**
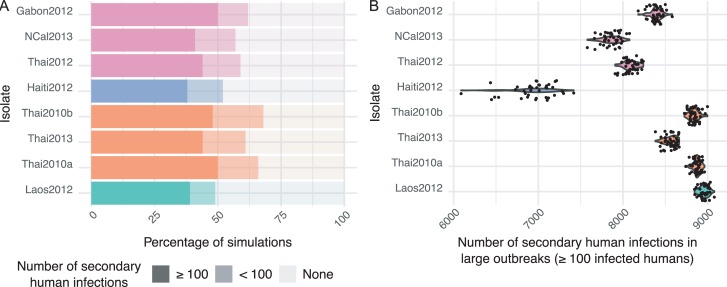
Simulated effect of variation in mosquito infection dynamics on the risk and magnitude of dengue outbreaks. A stochastic agent-based model was run 100 times for each combination of mean values of the three parameters (*K*, *B*, *M*) describing the kinetics of systemic mosquito infection. Other parameters, such as relative mosquito density, biting rate and human viral dynamics were held constant. Panel (A) shows the proportion of simulations that resulted in ≥100, <100 and no secondary human infections using the *K*, *B* and *M* values empirically measured for the eight DENV isolates. Panel (B) shows the total number of humans that became infected during large-scale dengue outbreaks (≥100 secondary human infections) using the empirical *K*, *B* and *M* values of the eight DENV isolates. In both panels, isolates are color-coded according to their AIC-based group of systemic mosquito infection kinetics.

Based on the empirical values of their parameter combinations, the risk and magnitude of dengue epidemics varied among the eight DENV isolates (Fig 4A and 4B). The probability that at least 1 secondary human infection occurred ranged from to 49% to 68%, and the probability of a large-scale dengue outbreak (≥100 secondary human infections) ranged from 38% to 50% among isolates (Fig 4A). During large-scale outbreaks, the mean proportion of the human population that became infected ranged from 69% to 90% among isolates (Fig 4B). In particular, the Haiti2012 isolate, which had the longest lag time (*M*) value, consistently led to a smaller outbreak size than all other isolates (Fig 4B). Overall, the probability and size of dengue outbreaks tended to be more similar among isolates from the same AIC-based group of infection kinetics described above. Differences between DENV isolates did not change significantly when EIP was modeled by a continuous 3-parameter function in individual mosquitoes or by a threshold function with variable EIP among mosquitoes (S1 File). However, when a single EIP value was assigned to the entire mosquito population it resulted in significant discrepancies between the simulations outputs (S1 File).

## Discussion

Building on earlier studies that showed EIP variation between DENV genotypes [23] and possibly serotypes [19, 20, 25], we have demonstrated that viral genetic variation in the kinetics of mosquito infection may contribute to heterogeneity in DENV transmission dynamics. Such temporal variation in the transmission process has generally been ignored in models of mosquito-borne pathogen epidemiology [9], with the exception of temperature-dependent models [10–12]. We experimentally compared field-derived DENV isolates representing the worldwide genetic diversity of recently circulating strains. We found significant variation in the dynamics of systemic infection of *Ae*. *aegypti* among the DENV isolates, which was poorly predicted by midgut infection patterns. Using large-scale simulations with a stochastic agent-based model, we illustrated how the empirical variation in systemic infection dynamics may translate into sizeable differences in epidemiological outcome (risk and magnitude of dengue outbreak in the human population).

While previous studies established a link between empirical differences in EIP duration and epidemiological fitness of arbovirus strains [23, 26], the effect of EIP on transmission has typically been extrapolated using a single value of EIP for the entire mosquito population. Our study went one step further by considering EIP as a quantitative trait following a population distribution [27]. Our results support the conclusion that the classic approach of measuring EIP as a ‘threshold’ time point at which the first mosquitoes become infectious is likely misleading for a mosquito population (S1 File). We used the cumulative proportion of infectious mosquitoes over time to infer the population distribution of lag time values (our proxy for EIP). Our results confirm that the distribution of lag time values cannot be described with a fixed threshold model because it derives from the sigmoid shape of a logistic function [28]. Furthermore, the inferred distribution of individual EIP values had a different shape between DENV isolates, adding another layer of complexity for comparing the epidemiological and evolutionary potential of different strains. Thus, our results lead to the conclusion that EIP is a quantitative trait that cannot be encapsulated in a single value for a given set of conditions. Although our general recommendation for future modeling studies is to use a full 3-parameter model whenever possible, EIP_50_ can be considered as the most relevant single parameter because *M* (half of the maximum proportion of infected mosquitoes with a systemic infection) was the most influential in our model.

It is well established that the duration of DENV EIP can be influenced by environmental factors such as temperature [12, 20, 29]. Recently, Ye and colleagues used a breeding design to demonstrate that the duration of DENV EIP is also influenced by mosquito genes [30]. Genetic variation is a prerequisite for the evolution of a trait, but it is unlikely that natural selection would act on mosquito populations to drive evolution of DENV EIP. This is because DENV infection in natural *Ae*. *aegypti* populations is rare [31] and does not significantly affect mosquito lifespan [32]. Conversely, variation in EIP duration among DENV strains could fuel the evolution of a shorter EIP. Under field conditions, a significant proportion of adult mosquitoes are expected to die before they are capable of transmitting virus. A shorter EIP would confer virus variants a significant evolutionary advantage by increasing their probability of transmission. However, DENV transmission rarely occurs before at least 7 days of EIP in *Ae*. *aegypti* [12, 19, 20, 29]. Unlike other arboviruses with an EIP that can be as short as 2–3 days [33, 34], the longer duration of DENV EIP is presumably evolutionarily constrained. Results from the present study indicate that evolution of a shorter DENV EIP is not restricted by a lack of standing genetic variation for this trait among virus strains.

Despite the evidence for genetic variation among virus variants, we did not detect any association between viral nucleotide polymorphisms and the parameters describing the kinetics of systemic mosquito infection. There was no consistent association between kinetics and DENV genotypes. For example, a 2012 isolate from Thailand belonging to genotype I displayed the longest lag time and a slow-disseminating profile. This isolate was phylogenetically closest to a 2012 isolate from Laos within genotype I, which displayed a fast-disseminating profile. Small nucleotide differences in the genome sequence can disproportionately affect DENV replication by modifying the secondary structure of the viral RNA molecule [35], which may contribute to large phenotypic differences between closely related viruses. However, the most likely explanation for the lack of genotype-phenotype association is that our study was underpowered for a genetic association analysis due to the small number of isolates examined.

We used the kinetics of systemic mosquito infection as a proxy for EIP dynamics due to the logistical and ethical constraints of experimentally measuring mosquito-to-human DENV transmission. We assumed that the virus presence in mosquito head tissues indicated the potential for onward transmission based on evidence that DENV is detected a few days earlier in head tissues than in salivary glands [36], and lack of evidence for a salivary gland escape barrier in *Ae*. *aegypti* [37]. However, it is possible that our measurements of EIP duration based on the lag time of systemic infection are underestimates. The probability of detecting DENV in *Ae*. *aegypti* saliva is positively correlated with the infectious titer of disseminated virus [17]. Therefore, we cannot exclude that some mosquitoes had virus titers in their head tissues that were high enough to be detected, but too low to result in virus release in saliva. The extent of underestimation is unknown but should not change the interpretation of differences between strains under the assumption that this underestimation is proportionate. Another limitation of our study is that we used a very narrow range of infectious doses to orally challenge the mosquitoes. Infectious dose is one of the most powerful determinants of DENV infection probability and EIP duration in *Ae*. *aegypti* [22, 38, 39]. It is therefore possible that the parameter estimates of mosquito infection kinetics would have been different if we had used a larger range of infectious doses. Finally, we used a minimal number of cell culture passages to produce the viral stocks, which may have promoted adaptation to mosquito cells. However, we know of no evidence that adaptation to mosquito cell culture would translate into differences in viral dissemination *in vivo*.

One important aspect of our results is that we evaluated the epidemiological significance of the observed variation in transmission kinetics among DENV isolates. We used mathematical modeling to incorporate variation in the dynamics of DENV transmission by mosquitoes into a relevant epidemiological framework. We implemented an agent-based model that included stochasticity for different parameters, such as the daily survival rate of mosquitoes and the probability of human-mosquito contact. Importantly, our model considered intra-host infection dynamics, both in humans and mosquitoes. Consistent with results from deterministic Ross-MacDonald models of vector-borne pathogen transmission [8, 40], our simulations indicated that EIP duration was the most influential factor underlying DENV transmission dynamics. In particular, EIP duration had a stronger influence than the maximum proportion of infectious mosquitoes on both the probability and the size of dengue outbreaks. We emphasize that these differences in transmission dynamics depend on the validity of our modeling assumptions. For instance, there could be other differences between DENV strains, such as human viral dynamics, that could counteract or modulate the effects observed in our model.

In conclusion, this study illustrated the power of combining empirical data obtained from experiments *in vivo* with epidemiological modeling to unravel the determinants of vector-borne pathogen transmission dynamics. DENV transmission dynamics are characterized by heterogeneities resulting from individual, temporal and spatial variabilities that are often difficult to identify [4–7]. We have shown that variability in mosquito infection dynamics exists among arbovirus strains and is epidemiologically meaningful. Such quantitative dissection of transmission dynamics contributes to improve our understanding of heterogeneities in arbovirus epidemiology.

## Materials and methods

### Experimental approach

#### Ethics statement

The Institut Pasteur animal facility has received accreditation from the French Ministry of Agriculture to perform experiments on live animals in compliance with the French and European regulations on care and protection of laboratory animals. This study was approved by the Institutional Animal Care and Use Committee at Institut Pasteur under protocol number 2015–0032.

#### Dengue virus isolates

Eight DENV type 1 isolates derived from human sera were selected from the collection of the French National Reference Center for Arboviruses based on their geographical origin and year of isolation in order to represent the worldwide viral genetic diversity of recently circulating strains (Table 1). Informed consent of the patients was not necessary because virus isolated in laboratory cell culture is no longer considered a human sample. Prior to their use in mosquito experimental infections, DENV isolates were amplified 2–6 times in C6/36 (*Aedes albopictus*) cells. Virus stock was prepared as previously described [27]. Virus titration was performed by focus-forming assay (FFA) in C6/36 cells after 5 days of incubation at 28°C, as previously described [27].

#### Experimental mosquito infections

Experiments were carried out with wild-type *Ae*. *aegypti* mosquitoes derived from a field population originally sampled in the Muang District of Kamphaeng Phet Province, Thailand and took place within 10 generations of colonization. Mosquitoes were maintained and orally exposed to DENV as previously described [27]. Briefly, five- to seven-day-old females deprived of sucrose solution for 24 hours were allowed to feed for 15 min from a membrane feeding system (Hemotek) that contained an artificial infectious blood meal maintained at 37°C. The infectious blood meal consisted of two volumes of washed rabbit erythrocytes and one volume of pre-diluted viral stock, supplemented with 10 mM adenosine triphosphate as a phagostimulant. The viral stock was pre-diluted in order to standardize the infectious dose among the DENV isolates. The infectious dose was chosen to result in an expected infection rate of 50–100%. The same infectious blood meal was used to expose three sequential batches of mosquitoes during a total of 45 min. An aliquot of the artificial blood meal was collected immediately prior and after blood feeding and stored at -80°C for later titration by FFA. The mean (± SD) log_10_-transformed viral titer dropped from 5.73 ± 0.13 to 5.46 ± 0.26 FFU/ml during the 45-min feeding period. This difference was statistically significant (t test: *p* = 0.0093) so the average of the two measurements for each isolate was used in all subsequent analyses. After virus exposure, fully engorged females were transferred to 1-pint cardboard containers and maintained under controlled conditions as described above with a maximum of 30 females per container.

#### DENV detection

The presence of a midgut and/or systemic DENV infection was determined on day 4, 6, 8, 12 and 18 post virus exposure. An infected body indicates a midgut infection, and an infected head indicates a systemic (disseminated) infection. Batches of mosquitoes were freeze-killed at each time point and their heads and bodies were separated and stored at -80°C until processing. Virus detection was performed for all mosquito bodies and for the heads of all mosquitoes with a DENV-positive body. The majority of samples were tested using a qualitative version of the FFA [27]. The qualitative version of the FFA is identical to the regular FFA except that it only tests the undiluted sample and therefore does not allow end-point titration. For a small subset of samples (S2 File) the FFA result was not reliable because of fungal contamination of the well. These undetermined samples were re-tested by two-step reverse transcription polymerase chain reaction (RT-PCR) detection of DENV RNA as previously described [27].

#### Virus sequencing

The full-length genomes of two of the DENV isolates (Thai2010a and Thai2010b) were already sequenced from a previous study [41] and are available in GenBank under accession numbers HG316481 and HG316482, respectively. The full-length genome sequences of the six other DENV isolates of the study were obtained by high-throughput sequencing. Total RNA was extracted from cell culture supernatant using QIAamp Viral RNA Mini Kit (Qiagen), DNAse treated (Turbo DNAse, Life Technologies) and purified with magnetic beads (Beckman Coulter). Purified RNA was then reverse transcribed using Transcriptor High Fidelity cDNA Synthesis Kit and random hexamers (Roche Applied Science, Penzberg, Germany). Second DNA strand was synthetized in a single reaction with *E*. *coli* DNA ligase (NEB), *E*. *coli* DNA polymerase I (NEB), *E*. *coli* RNAse H (NEB) in second strand synthesis buffer (NEB). Resulting dsDNA was purified with magnetic beads (Agencourt AMPure XP, Beckman Coulter) and its concentration was measured by fluorometric quantification (Quant-iT PicoGreen dsDNA, Invitrogen). Sequencing libraries were prepared using Nextera XT DNA Library Preparation Kit (Illumina), multiplexed and sequenced in single-end on an Illumina NextSeq 500 platform using a mid-output 150-cycle v2 kit (Illumina).

After demultiplexing, trimmomatic v0.33 [42] was used to discard reads shorter than 32 nucleotides, filter out Illumina adaptor sequences, remove leading and trailing low quality bases and trim reads when the average quality per base dropped below 15 on a 4-base-wide sliding window. Full DENV genomes were reconstructed by *de novo* assembly using Ray v2.0.0 [43]. The largest contig was extended in 3’ and 5’ using the full genome sequence of the closest BLAST hit. This chimeric construct was used as the reference to re-map all the reads using Bowtie 2 v2.1.0 [44] and correct the 3’ and 5’ ends. The alignment file was converted, sorted and indexed using Samtools v0.1.19 [45]. Coverage and sequencing depth was assessed for each sample using bedtools v2.17.0 [46]. Single nucleotide variants and their frequency were called using LoFreq* v2.1.1 [47] and used to correct the chimeric construct. Only nucleotides with >10X coverage were conserved for generating the consensus sequence. The full-length genomes of the six newly sequenced DENV isolates were deposited in GenBank under accessions numbers MG877552 (Laos2012), MG877553 (Haiti2012), MG877554 (Thai2012), MG877555 (Ncal2013), MG877556 (Thai2013), and MG877557 (Gabon2012).

#### Phylogenetic analyses

A background set of 51 full-length DENV genome sequences was obtained from GenBank to represent the different genotypes of DENV type 1 [48]. Genome sequences were aligned using ClustalW v.2.0.12 [49] and analyzed with RAxML v.8.2.10 [50] to generate the best-scoring maximum-likelihood (ML) tree of 10 runs with 100 thorough bootstrap replicates. The GTR + I + G nucleotide substitution model was chosen based on the lowest Bayesian Information Criterion (BIC) value using the JModelTest 2 software [51]. The same procedure was applied to the eight DENV isolates. Phylogenetic trees were visualized using FigTree v.1.4.3 [52] and the ggtree R package [53]. Phylogenetic comparative analysis of continuous traits was performed with the phyloSignal function of the phylosignal R package [54] using the Abouheif's Cmean, Moran's I, Blomberg's K and Pagel's Lambda methods [55].

#### Empirical data analysis

A global likelihood function was used to model the probabilities of systemic mosquito infection over time for each virus isolate. Probabilities of systemic infection at each time point post virus exposure were estimated with a 3-parameter logistic model (Eq 1) assuming a binomial distribution of systemic infection status.

$$f\left(t,\left(K,B,M\right)\right)=K/(1+e^{-B\left(t-M\right)})$$ Eq 1

Based on this function, the probability of systemic infection at a given time point (*t*) is governed by three parameters. *K* is the saturation level and represents the maximum proportion of mosquitoes with a systemic infection. *B* is the slope factor and represents the maximum value of the slope during the exponential phase of the cumulative function, scaled by *K*. For easier biological interpretation, *B* was transformed into Δt, which corresponds to the time required to rise from 10% to 90% of the saturation level with the formula: Δt = ln(81)/*B* [56, 57]. *M* is the lag time and represents the time at which the absolute increase in cumulative proportion is maximal. For each isolate, the subplex R function [58] was used to provide the best estimates of the three parameters to maximize the global likelihood function (i.e., the sum of binomial probabilities at each time point post virus exposure). This method accounts for differences in sample size when estimating parameters values. The daily rate of new systemic infections was obtained by calculating the first derivative of the cumulative systemic infection function.

Comparison of systemic infection kinetics among DENV isolates was based on the Akaike Information Criterion (AIC). The global likelihood model was fitted to all 4,140 partitions of isolates. Isolates from one group were constrained to share the same parameter values. AIC values were calculated for all possible partitions of isolates with the same empirical data of systemic infection. The best isolate grouping was considered as the one with the lowest AIC score. An affinity matrix among isolates was derived from AIC values obtained from all grouping comparisons. Two isolates share a higher affinity if they are commonly grouped together in models with low AIC values. A hierarchical clustering analysis was performed on the affinity scores to reveal the extent of similarities of systemic infection kinetics among isolates.

The time-dependent effect of infectious dose (blood meal titer) on mosquito midgut infection and systemic infection was analyzed by logistic regression. Differences in mosquito infection over time were analyzed using hierarchical clustering with the Ward method based on the scaled proportion of infected mosquitoes at each time point post exposure. Pvclust [59] was used to calculate Approximately Unbiased (AU) *p* values for hierarchical clustering through multiscale bootstrap resampling using 1,000 bootstrap replicates. Genome-wide associations were tested by analysis of variance of the kinetics parameter values as a function of viral sequence polymorphisms (at the nucleotide and amino-acid levels). The null hypothesis was the lack of difference in parameter values between polymorphic variants at each locus. It was rejected when *p* values were <0.05 after Bonferroni correction for multiple testing. Geneious v.7.0.6 (http://www.geneious.com, [60]) was used to align and translate the viral genomic sequences. All statistical analyses were performed in the R statistical environment [61] and the R Markdown in provided as S3 File. Charts were generated with the ggplot2 package [62].

### Epidemiological modeling

#### Model structure overview

A stochastic agent-based model was developed in a closed human population to assess the influence of variation in the dynamics of systemic mosquito infection on dengue outbreaks. An agent-based model was chosen to achieve a more intuitive integration of the complex interplay of relatively simple rules within and between agents than would be possible with a mechanistic model. Starting with one infected human, this model encapsulates arbovirus transmission between susceptible mosquitoes and humans within a simulated environment. The model considers the influence of intra-host infection dynamics, both in humans and mosquitoes, on virus transmission probability during human-mosquito contacts. The model was run for several combinations of the three parameters (*K*, *B* and *M*) that were used above to describe the kinetics of systemic mosquito infection. The main epidemiological outcome was the total number of secondary human infections. The entities of the model were individual humans and mosquitoes. The variable states of humans were uninfected/infected. The variable states of mosquitoes were alive/dead and uninfected/infected. The time step of the model was one day. The values assigned to the model variables are shown in S1 Table.

#### Human and vector populations

Human and mosquito population sizes were set as constant for each simulation. The model was populated with 10,000 humans and 30,000 mosquitoes. No birth, death or migration was allowed for humans. At each time point, daily mosquito deaths were compensated by the birth of uninfected mosquitoes. The model assumed homogeneous mixing of humans and mosquitoes, allowing any mosquito to bite any human with equal probability. At each time point in the simulation, a subset of the mosquito population was randomly chosen based on the daily feeding rate (S1 Table) to bite randomly chosen humans. The maximal number of daily bites of an individual mosquito was limited to one, whereas several mosquitoes could bite an individual human in one day.

#### Transmission event

The model only considered arbovirus transmission (*i*) from an infected human to uninfected vectors and (*ii*) from an infected vector to susceptible humans. The model ignored vertical transmission from an infected female to its offspring because it is rare both in humans [63] and in mosquitoes [64] and can be considered as epidemiologically insignificant. The model assumed that humans acquired protective immunity from a previous infection, whereas infection clearance was not allowed in mosquitoes. No superinfection events were allowed in mosquitoes and humans that would change infection dynamics.

#### Human-to-mosquito transmission

Dynamics of human-to-mosquito transmission probability was modeled using a 5-parameter equation (Eq 2) where the transmission probability *pvert* is linked to time since infection (*t*), the maximum probability of transmission (*K*_*human*_), time when 50% of *K*_*human*_ value is attained during ascending probability (*Mγ*), slope of the ascending curve (*Bγ*), time when 50% of *K*_*human*_ value is attained during descending probability (*Mδ*), and slope of the descending curve (*Bδ*).

$$pvert\left(t\right)=K_{human}/\left(1+e^{\left(-B\gamma \times \left(t-M\gamma \right)\right)}\right)-K_{human}⁄\left(1+e^{\left(-B\delta \times \left(t-M\delta \right)\right)}\right)$$ Eq 2

For each individual human, values for these five parameters were drawn from a probability distribution, of which specific parameters are provided in S1 Table. *Mγ*, *Bγ*, and *Bδ* were drawn from a normal probability distribution, *δ* was drawn from a truncated normal probability distribution (to avoid values <*Mγ*), and *K*_*human*_ was drawn from a beta distribution. Distributions of each parameter were set based on previously published data on DENV infection dynamics in humans (S1 Table). By reanalyzing classic studies in which DENV-infected human volunteers were experimentally exposed to mosquitoes [19, 25], Nishiura and Halstead inferred probabilities of successful transmission from humans to mosquitoes according to time of disease onset in humans [65]. The link between time since infection and transmission probability was obtained by transforming time of disease onset in humans into time post human infection based on the work of Chan and Johansson [66]. Using a meta-analysis, Chan and Johansson estimated that the average time between infection and onset of the symptoms (the intrinsic incubation period) was about 5.9 days for dengue. Eq 1 was fitted to the transformed data to estimate parameter values presented in S1 Table.

#### Mosquito-to-human transmission

Dynamics of mosquito-to-human transmission probability was modeled using the 3-parameter equation (Eq 1) described above. For each individual mosquito, values for the three parameters were drawn from a probability distribution (S1 Table). *M* was drawn from a normal probability distribution, *B* was drawn from a truncated normal distribution (to avoid values <0) and *K* was drawn from a beta distribution. Alternative modeling strategies are described in S1 File.

#### Implementation

The model was implemented in R version 3.4.0 using the packages foreach [67], doParallel [68], plyr [69], reshape2 [70], and data.table [71]. A total of 1,140 different parameter combinations for *K*, *B*, and *M* were run. Two sets of simulations were run. The first set aimed to explore the parameter space of *K*, *B*, and *M*. An arbitrary range of mean values was generated for each parameter by increasing from a realistic minimum value to a realistic maximum value in a stepwise fashion (the increment size is provided in S1 Table). A total of 1,140 different parameter combinations for *K*, *B*, and *M* were run, and each combination was run for 100 independent replicate simulations, yielding a total number of 114,000 simulations. The second set of simulations aimed to explore epidemiological outcomes for the eight empirically characterized DENV isolates. In these simulations, mean values of *K*, *B*, and *M* were set to their respective experimentally determined estimates, whereas standard deviation was the same as in the first set of simulations (S1 Table). Epidemics caused by each DENV isolate were examined in 100 independent replicate simulations. Each simulation was initiated with one infected human at *t* = 0, then each simulation was run for 400 days. The code of simulations is available at https://github.com/slequime/ArboEpiSim.

#### *In silico* data analysis

For each replicate of a given parameter combination, the total number of humans infected during the outbreak was recorded. Two outputs derived from the simulation data were examined: (*i*) the proportion of failures to initiate an outbreak and (*ii*) the mean number of secondary human infections across replicates. Failure to initiate an outbreak was defined as the lack of secondary infections over the length of the simulation following initial introduction of a single infected human. Failures to initiate an outbreak were filtered out before calculating the mean number of human infections. For each analysis, a stratified random splitting was performed on the data with the createDataPartition function from the caret R package [72] to obtain *training* and *test* subsets of the data with a balanced number of sample for each parameters combinations. The *training* set was composed of 70% of the data. We performed random forest regression with optimized parameter settings provided by the train function of the caret R package [72]. A total of 500 trees were used for both analyses with two and four variables or interaction between variables randomly tried at each split. The model included the proportion of failures to initiate an outbreak as the response variable and the three parameters describing systemic infection dynamics and their interaction as explanatory variables. The relative importance of parameters was determined according to their *IncNodePurity* value, which measures the quality of a split for each variable, averaged over all trees.

## Supporting information

S1 Fig Observed prevalence of mosquito midgut infection following oral exposure to DENV isolates. (A) Bar charts represent the proportion of mosquitoes with a DENV-infected midgut at several time points following oral exposure. Dashes represent the 95% confidence intervals of the proportions. Time-averaged proportions are shown on the right side of each chart. Midgut infection prevalence was significantly influenced by the time point (*p* = 6.6×10^−9^), and increased with uncontrolled variation in blood meal titers although not significantly so (*p* = 0.12). (B) The heat map represents midgut infection prevalence (using scaled values to standardize variation across time points) over time post DENV exposure for each isolate. Hierarchical clustering dendrograms are displayed on the left side of the heat map with Approximately Unbiased (AU) values (in %) computed by multiscale bootstrap resampling. A green rectangle highlights isolates groupings with AU values larger than 95%.
(EPS)

S2 Fig Pairwise correlations between the three parameters of systemic mosquito infection dynamics. For each isolate, the cumulative change in the proportion of mosquitoes with a systemic DENV infection over time post exposure was used to fit a 3-parameter logistic model. *K* is the saturation level and represents the maximum proportion of mosquitoes with a systemic infection. *B* is the slope factor and represents the maximum slope of the cumulative function scaled by *K*. *M* is the lag time and represents the time at which the absolute increase in cumulative proportion is maximal. Blue lines represent linear regressions and the gray areas are their confidence intervals. Spearman’s rank correlation coefficients and *p* values are indicated below each graph.
(EPS)

S3 Fig Statistical independence between parameter of systemic mosquito infection dynamics and phylogenetic relatedness among DENV isolates. (A) Scaled and centered parameters (*K*: saturation level; *B*: slope factor; *M*: lag time) describing the kinetics of systemic mosquito infection are mapped along the phylogeny of the 8 DENV isolates. The tree was constructed with a maximum-likelihood method implemented in RAxML v.8.2.10 [50] using the full-genome sequence of the isolates. The scale bar represents the number of substitutions per site. (B) Statistical independence between parameter values and phylogenetic relatedness among isolates was assessed using the Abouheif's Cmean, Moran's I, Blomberg's K and Pagel's Lambda methods implemented in the phylosignal R package [54]. The table displays *p* values obtained with each method.
(EPS)

S4 Fig Analyses of simulations outputs. (A) Density chart of the mean number of infected humans across simulation replicates for several combinations of *K* and *M* (with parameter *B* drawn from a uniform distribution). *K* and *M* were the two most influential parameters identified by random forest analysis performed on two independent simulation outputs: (B) the proportion of failures to initiate an outbreak and (C) the mean number of human infections across replicates. Overall, random forest analysis explained 88.9% and 96.6% of the variability in the proportion of failure to initiate an outbreak and in the mean number of human infections in successful outbreaks, respectively. In panels (B) and (C), upper charts show the correlation between observed and predicted values on the *test* data subset. Bottom charts show the relative importance of infection kinetics parameters, with higher *IncNode Purity* values meaning greater importance.
(EPS)

S5 Fig Identification of the best-fit model of systemic mosquito infection kinetics among all possible isolate groupings. A logistic 3-parameter model was fitted to all permutations of isolates, with isolates from the same group being forced to share the same parameters. AIC values for each isolate grouping are represented as a function of the number of groups in the model. The inset shows AIC values for all isolate groupings. The main graph shows the isolate groupings in the lower range of AIC values. The lowest AIC value is indicated by a green circle and represents the isolate grouping that maximizes the probability function shown in Fig 3A.
(TIF)

S1 Table Central values used for each parameter in the agent-based model. *Parameters drawn from a beta distribution. **Parameters drawn from a truncated normal distribution to avoid non-existing values.
(DOCX)

S1 File Comparison of simulation outputs between EIP modeling strategies. Simulations results are presented for the eight DENV isolates when EIP was modeled by a 3-parameter equation, a threshold effect with variable EIP, or a fixed EIP value (EIP_50_ or EIP_10_) for the entire mosquito population.
(PDF)

S2 File Raw empirical data from this study. Each line represents an individual mosquito and the columns are the experimental conditions and phenotypes. From left to right, the columns indicate the DENV isolate, number of days post infectious blood meal, body infection status, head infection status, infection round (out of three sequential 15-min rounds of exposure), experiment (out of two separate experiments), blood meal titer (in log_10_ FFU/ml), number of prior passages in C6/36 cells, country of origin, date of isolation, and DENV-1 genotype. The last column identifies the small number of samples that were tested by PCR instead of FFA.
(TXT)

S3 File R Markdown document of the study. The file provides code lines used to analyze the experimental data and perform data visualization. The R code lines only refer to the procedure of systemic infection modeling and the AIC-based method to compare systemic infection across isolates. This document can be opened in Rstudio [73] with S2 File as the input file.
(RMD)

S4 File Simulated effect of mosquito infection kinetics on dengue outbreaks. The interactive bubble plot represents the probability and size of dengue outbreaks across simulation replicates as a function of the three parameters (*K*, *B*, *M*) describing the kinetics of systemic mosquito infection. The size of the bubble is proportional to the percentage of successful outbreaks (>1 infected human). The color of the bubble represents the log_10_-transformed mean number of infected humans during successful outbreaks. Hover text indicates the percentage of successful outbreaks, the mean number of infected humans during successful outbreaks, and the parameter values when the pointing device is moved over a bubble. This chart was made with Plotly for R v. 4.7.1 [74] and can be viewed in most web browsers.
(HTML)
