# Supplementary figures and images for: Epidemiological significance of dengue virus genetic variation in mosquito infection dynamics
Source: PLoS Pathog. 2018 Jul 13;14(7):e1007187. doi: 10.1371/journal.ppat.1007187 (PMC6059494; doi:10.1371/journal.ppat.1007187)

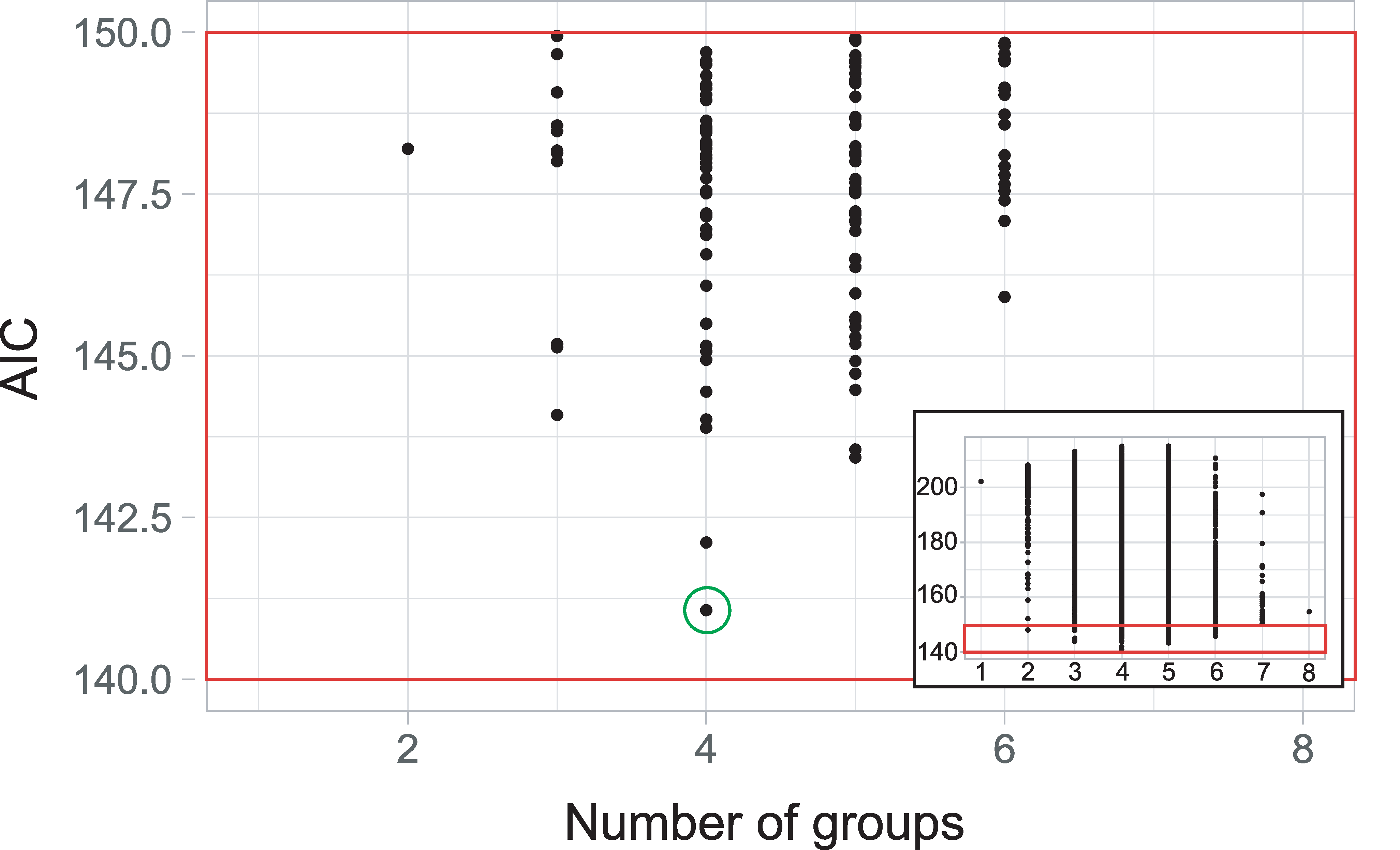

Supplement: S5 Fig — A logistic 3-parameter model was fitted to all permutations of isolates, with isolates from the same group being forced to share the same parameters. AIC values for each isolate grouping are represented as a function of the number of groups in the model. The inset shows AIC values for all isolate groupings. The main graph shows the isolate groupings in the lower range of AIC values. The lowest AIC value is indicated by a green circle and represents the isolate grouping that maximizes the probability function shown in Fig 3A. (TIF) [file ppat.1007187.s005.tif]
