# Supplementary material for: Epidemiological significance of dengue virus genetic variation in mosquito infection dynamics
Source: PLoS Pathog. 2018 Jul 13;14(7):e1007187. doi: 10.1371/journal.ppat.1007187 (PMC6059494; doi:10.1371/journal.ppat.1007187)
Supplement: S1 Table — *Parameters drawn from a beta distribution. **Parameters drawn from a truncated normal distribution to avoid non-existing values. (DOCX) [file ppat.1007187.s006.docx]

| **Notation** | **Description** | **Value** | **Reference** |
| --- | --- | --- | --- |
| *General simulation parameters* | | | |
|  | Number of humans in the population | 10,000 |  |
|  | Relative density of mosquitoes in the population | 3 | [1] |
|  | Number of initially infected mosquitoes | 0 |  |
|  | Number of initially infected humans | 1 |  |
| *Entomological parameters* | | | |
|  | Mosquito daily survival probability | 0.85 | [1] |
|  | Mosquito daily biting probability | 0.3 | [2] |
| *Human* *infection dynamics* | | | |
| *K_human_* | Maximum probability of transmission (mean) | 1* | [3] |
|  | Standard deviation of *p_max_* | 0.04 |  |
| *Mγ* | Time when 50% of *p_max_* value is attained during ascending probability (mean) | 4.844 days |  |
|  | Standard deviation of *γ* | 0.5 |  |
| *Bγ* | Slope of the ascending curve (mean) | 2.536 |  |
|  | Standard deviation of *s_γ_* | 0.2 |  |
| *Mδ* | Time when 50% of *p_max_* value is attained during descending probability (mean) | 8.392 days** |  |
|  | Standard deviation of *δ* | 0.5 |  |
| *Bδ* | Slope of the descending curve (mean) | 4.038 |  |
|  | Standard deviation of *s_δ_* | 0.2 |  |
| *Mosquito infection dynamics* | | | |
| *K* | Maximum probability of transmission (mean) | 0.5 – 1* step: 0.1 | This study |
|  | Standard deviation of *K* | 0.04 |  |
| *B* | Slope factor | 0.4 – 4** step: 0.2 |  |
|  | Standard deviation of *B* | 0.2 |  |
| *M* | Time when 50% of *K* value is attained | 3 – 11 days step: 1 |  |
|  | Standard deviation of *M* | 0.5 |  |

**References**

1. Favier C, Schmit D, Muller-Graf CD, Cazelles B, Degallier N, Mondet B, et al. Influence of spatial heterogeneity on an emerging infectious disease: the case of dengue epidemics. Proc Biol Sci. 2005;272(1568):1171-7.

2. Andraud M, Hens N, Marais C, Beutels P. Dynamic epidemiological models for dengue transmission: a systematic review of structural approaches. PLoS One. 2012;7(11):e49085.

3. Nishiura H, Halstead SB. Natural history of dengue virus (DENV)-1 and DENV-4 infections: reanalysis of classic studies. J Infect Dis. 2007;195(7):1007-13.
