# Supplementary material for: Epidemiological significance of dengue virus genetic variation in mosquito infection dynamics
Source: PLoS Pathog. 2018 Jul 13;14(7):e1007187. doi: 10.1371/journal.ppat.1007187 (PMC6059494; doi:10.1371/journal.ppat.1007187)
Supplement: S1 File — Simulations results are presented for the eight DENV isolates when EIP was modeled by a 3-parameter equation, a threshold effect with variable EIP, or a fixed EIP value (EIP50 or EIP10) for the entire mosquito population. (PDF) [file ppat.1007187.s007.pdf]

Comparison between EIP modeling strategies

In our agent-based model, mosquito-to-human transmission events were determined for each host-vector contact based on a probability distribution as a function of time after mosquito infection. This probability distribution was inferred from the empirical data obtained from the eight DENV isolates of the study. We used the empirical cumulative proportion of infectious mosquitoes over time as a proxy for mosquito-to-human transmission probability distribution. Due to the sacrificial nature of the assay, this cumulative function derives from the distribution of EIP values observed at the mosquito population level, but does not necessarily represent transmission probabilities at the individual mosquito level. Therefore, it is unknown whether an individual mosquito transmits with 0% probability prior to its EIP value and with 100% probability after its EIP (a threshold effect), or whether transmission probability is also a continuous function of time at the individual mosquito level. In the main part of the study, we chose to model individual transmission probability as a continuous function of time estimated by the cumulative function at the population level. In this supplementary file, we explore alternative ways to model EIP based on a threshold effect. We compared three EIP modeling strategies, keeping the rest of the agent-based model identical:

3-parameter equation. For each individual mosquito the probability of virus transmission is directly determined by the 3-parameter logistic function (K, B, M) inferred from the empirical data. In this case the cumulative function is used to model the EIP dynamics of individual mosquitoes. This model was used in the main study.

Threshold with variable EIP. For each individual mosquito, an EIP value is drawn from the empirical distribution of EIP values (mean = M, scale = $1/B$). For this mosquito, the probability to transmit equals zero prior to its EIP value, and K after its EIP value. The EIP

value thus acts as a threshold time point at the individual mosquito level but this threshold value varies among mosquitoes.

Threshold with fixed EIP. For all mosquitoes, the probability to transmit equals zero prior to a fixed EIP value, and *K* after the fixed EIP value. The EIP value thus acts as a collective threshold time point and does not vary among mosquitoes. The fixed EIP value was derived from the empirical data, based on the time when 50% of mosquitoes (EIP_{50}) or 10% of mosquitoes (EIP_{10}) were able to transmit the virus. Fixed EIP_{50} and EIP_{10} models simulate experimental designs in which a single EIP value is used for an entire mosquito population.

Like in the main part of the study, each simulation was initiated with one infected human introduced into a population of 10,000 humans and 30,000 mosquitoes. Each simulation was run for 400 days and a total of 100 replicate runs were performed for each of the eight DENV isolates. For each simulation, the total number of infected humans during the outbreak was recorded. We analyzed the proportion of outbreaks of differing size (no secondary infection, <100 infected humans and ≥100 infected humans) and the total number of secondary human infections in large outbreaks (≥100 infected humans). The effect of the EIP model on differences between isolates was measured by the statistical significance of the model x isolate interaction in an ordinal logistic regression of the proportion of outbreaks of differing size (*polr* function in R) and an analysis of variance of the total number of secondary infections in large-scale outbreaks.

The simulations results for each model are shown in Fig A below. For both simulations outputs analyzed (proportions of outbreaks of differing size and total of secondary infections in large-scale outbreaks), there was no statistically significant difference ($p > 0.05$) between the EIP model based on the 3-parameter equation and the EIP model based on the threshold time point with variable EIP. In contrast, there was a significant main effect of the fixed EIP_{10} model relative to the 3-parameter equation model ($p = 0.0042$) on the proportion of outbreaks

of differing size. There was a significant effect of the model x isolate interaction on the proportion of outbreaks of differing size between models using the 3-parameter equation and the fixed EIP_{50} (p = 0.0376). There was also a significant effect of the model x isolate interaction on the total number of human infections in large-scale outbreaks between models using the 3-parameter equation and the fixed EIP_{50} or EIP_{10} values ($p < 0.0001$).

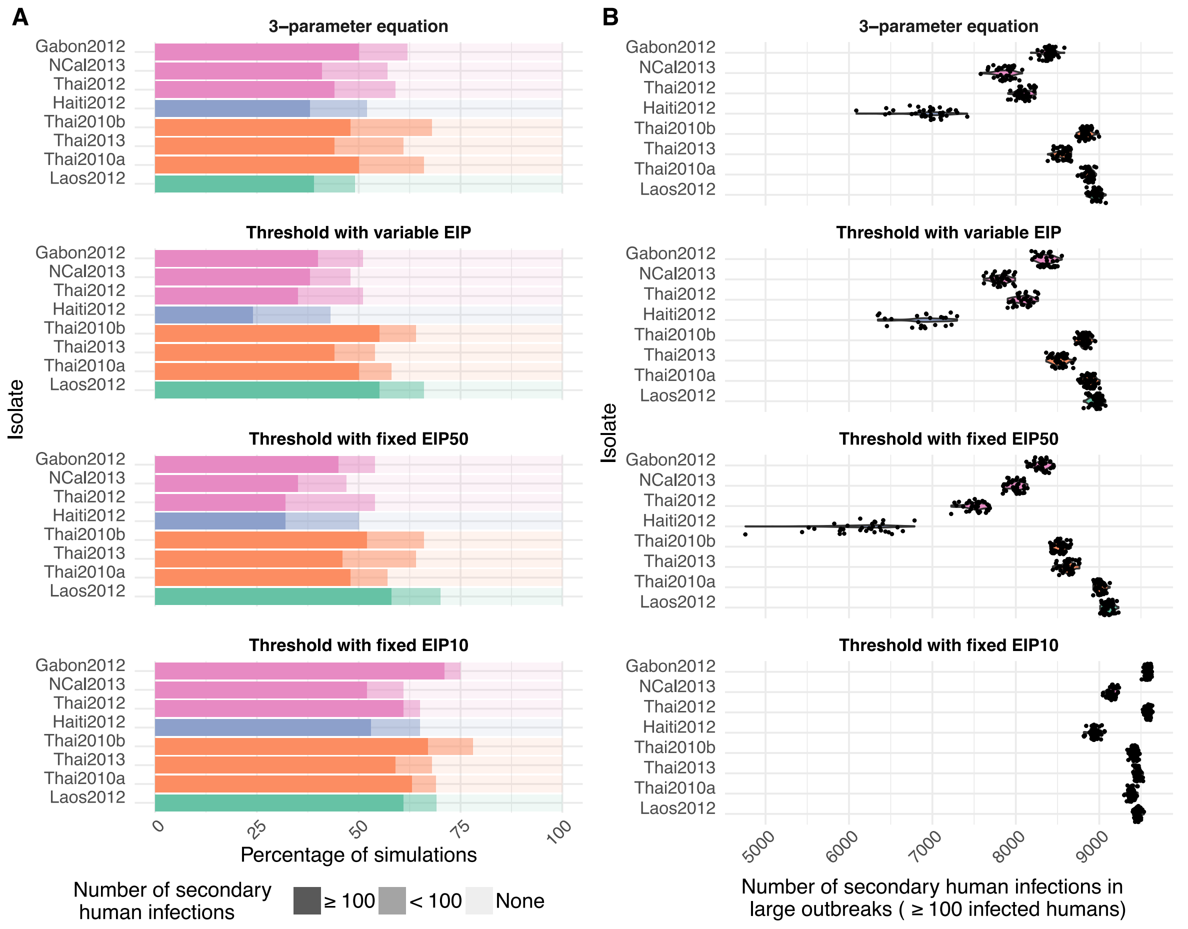

Figure A. Simulated effect of variation in mosquito infection dynamics on the risk and magnitude of dengue outbreaks using different EIP models. A stochastic agent-based model was run using different models to describe the kinetics of systemic infection in individual mosquitoes (3-parameter equation, threshold with variable EIP, and fixed EIP_{10} or EIP_{50}) based on the empirical values obtained for eight DENV isolates. Other parameters, such as relative mosquito density, biting rate and human viral dynamics were held constant. Panel (A) shows the proportion of simulations that resulted in ≥100, <100 or no secondary infection. Panel (B) shows the total number of humans that became infected during large-scale dengue outbreaks (≥100 human infections). In both panels, DENV isolates are color-coded according to their AIC-based group of systemic mosquito infection kinetics.

Overall, simulations using alternative strategies to model EIP showed that using the 3-parameter equation or a threshold with variable EIP lead to similar epidemiological outcomes for the eight DENV isolates of the study. Conversely, using a fixed EIP value for the entire mosquito population resulted in inconsistent epidemiological outcomes among the DENV isolates. The fixed EIP_{10} model, in particular, largely overestimated the proportion of large-scale outbreaks and total number of secondary cases relative to the other models. These results support the conclusion that the classic approach of measuring EIP as a unique threshold time point for a whole mosquito population is likely misleading.
